# Introduction: priority setting in global health

**DOI:** 10.1186/s12962-018-0115-x

**Published:** 2018-11-09

**Authors:** David E. Bloom, Daniel Cadarette, Rashmi Dayalu, Jessica Sullivan

**Affiliations:** Department of Global Health and Population, Harvard T.H. Chan School of Public Health, Boston, USA

The process of setting priorities for social spending is inherently complex. It requires, in general, taking account of heterogeneity in preferences and expectations over a wide range of options and outcomes. It may involve multiple stakeholders, each with different and not-necessarily aligned interests, within or across different sectors. Priority setting processes can also incorporate a varying range of subjective/qualitative and objective/quantitative considerations. Resource-allocation decisions can be shaped by institutional requirements or strictures and might also be driven by political expediency and the desire to build popular support.

Multi-criteria decision analysis (MCDA) is an approach that supports priority setting “by taking explicit account of multiple criteria when helping individuals or groups explore decisions that matter” [1]. Researchers at the Harvard T.H. Chan School of Public Health hosted a *Priority Setting in Global Health* symposium in Cambridge, Massachusetts on October 5–6, 2016, with a special focus on exploring MCDA’s strengths and identifying practical solutions to its limitations. This symposium brought together under one roof some of the world’s leading experts on MCDA and global health. Symposium participants included co-chairs of the International Society for Pharmacoeconomics and Outcomes Research (ISPOR) MCDA Emerging Good Practices Task Force, developers and users of MCDA instruments in various contexts (e.g., the EVIDEM framework and the SMART Vaccines tool), academics and researchers from a variety of disciplines (including biotechnology, economics, epidemiology, medical ethics, and medicine), and political representatives from around the world. This special issue is a collection of cutting-edge MCDA research, reviews, and commentaries built on the illuminating presentations and comments offered by symposium participants. (See Tables 1 and 2 for the symposium agenda and the full participant list).

**Table 1:** Priority setting in global health, October 2016, Cambridge, MA: Agenda

Session type	Session title	Session participant(s)
Wed, October 5, 2016		
Keynote	MCDA: a new paradigm for healthcare decision making?	Mireille Goetghebeur
Thursday, October 6, 2016		
Introduction	Introductory remarks	David Bloom
Presentation	HTA in Latin America: a tool for explicit priority setting in Colombia	Hector Castro
Presentation	Strategic planning tools for preparedness	Guru Madhavan Charles Phelps
Presentation	SMART Vaccines 2.0: piloting further development of a multi-criteria decision analysis tool	Bruce Gellin Stacey Knobler
Presentation	Antares health priorities matrix: application in Waikato District, New Zealand	Rashmi Dayalu
Presentation	MCDA: do not provide a mathematical solution to what really is an ethical problem	Rob Baltussen
Panel discussion	Considerations for development of MCDA tools	Ole Norheim Kevin Marsh Cristian Baeza Tessa Tan-Torres Edejer Mark Jit
Panel discussion	Considerations for applications of MCDA tools	Michael Watson Kalipso Chalkidou Gillian SteelFisher Mahlet Kifle Habtemariam
Summary	Wrap-up & closing remarks	Guru Madhavan Ole Norheim David Bloom

**Table 2:** Priority setting in global health, October 2016, Cambridge, MA: Participants

Last name	First name	Title	Affiliation
Baeza	Cristian	Excecutive Director	Center for Healthy Development
Baltussen	Rob	Professor of Global Health Economics	Radboud University Nijmegen
Bloom	David	Professor of Economics and Demography	Harvard T.H. Chan School of Public Health
Cadarette	Daniel	Research Assistant	Harvard T.H. Chan School of Public Health
Canning	David	Professor of Economics and International Health	Harvard T.H. Chan School of Public Health
Castro	Héctor	Director of Medicines & Health Technologies	Ministry of Health and Social Protection, Colombia
Chalkidou	Kalipso	Director, Global Health and Development Group	Institute of Global Health Innovation, Imperial College London
Daniels	Norman	Professor of Ethics and Population Health	Harvard T.H. Chan School of Public Health
Dayalu	Rashmi	Research Assistant	Harvard T.H. Chan School of Public Health
Edejer	Tessa Tan-Torres	Coordinator, Department of Health Financing and Governance	World Health Organization
Eyal	Nir	Associate Professor of Global Health and Population	Harvard T.H. Chan School of Public Health
Fan	Victoria	Assistant Professor	University of Hawai‘i
Fonseca	Elizabeth	Program Director for Population Health Management	Massachusetts General Hospital
Gellin	Bruce	Director of the National Vaccine Program Office	U.S. Department of Health & Human Services
Glass	Roger	Director	Fogarty International Center
Goetghebeur	Mireille	Adjunct Professor	University of Montreal
Hammitt	James	Professor of Economics and Decision Sciences	Harvard T.H. Chan School of Public Health
Hennis	Anselm	Director, Department of Noncommunicable Diseases and Mental Health	Pan American Health Organization
Holmboe	Dag	Founder	Klurig Analytics
James	Ralph	Executive Director, External Relations	Harvard Business School
Jit	Mark	Professor of Vaccine Epidemiology	London School of Hygiene and Tropical Medicine
Kachur	Patrick	Chief of the Malaria Branch	U.S. Centers for Disease Control and Prevention (CDC)
Khampang	Roongnapa	Researcher	Health Intervention and Technology Assessment Program (HITAP), Ministry of Public Health, Thailand
Kifle Habtemariam	Mahlet	Takemi Fellow	Harvard T.H. Chan School of Public Health
Knobler	Stacey	Scientific Program Director	Division of International Epidemiology and Population Studies (DIEPS), National Institutes of Health
Madhavan	Guru	Biomedical Engineer, Senior Policy Adviser	National Academies of Sciences, Engineering, and Medicine
Marsh	Kevin	Senior Research Leader	Evidera
Norheim	Ole	Adjunct Professor of Global Health and Population	Harvard T. H. Chan School of Public Health
Onarheim	Kristine Husøy	PhD Candidate	University of Bergen
Payne	Roslyn	President	Payne Family Foundation
Phelps	Charles	Provost Emeritus	University of Rochester
Ratcliffe	Amy	Director, Program Analytics	Population Services International
Reich	Michael	Professor of International Health Policy	Harvard T.H. Chan School of Public Health
Sevilla	J.P.	Research Associate	Harvard T.H. Chan School of Public Health
Smullin	Alix	Attorney	Good Neighbor Mediation Project
SteelFisher	Gillian	Senior Research Scientist	Harvard T.H. Chan School of Public Health
Sullivan	Jessica	Assistant Director of Research, Department of Global Health and Population	Harvard T.H. Chan School of Public Health
Thier	Samuel	Professor of Medicine and Health Care Policy, Emeritus	Harvard Medical School
Thokala	Praveen	Health Economics Modeler	University of Sheffield
Verguet	Stéphane	Assistant Professor of Global Health	Harvard T.H. Chan School of Public Health
Voorhoeve	Alex	Professor of Philosophy	London School of Economics and Political Science
Watson	Michael	Senior Vice President, Vaccines Partnerships & Health Impact	Moderna Therapeutics
Youngkong	Sitaporn	Faculty of Pharmacy	Mahidol University

Over the past decade, MCDA has increasingly been discussed and adapted to address the challenges of priority setting in global health. MCDA uses health and health technology impact data to rank a variety of decision alternatives in order of priority, based on multiple explicit criteria that are articulated, evaluated/scored for their impact, and weighted by relevant stakeholders. Proponents of MCDA believe that its three core strengths are its pragmatism, its basis in real-world evidence as well as the contextual preferences of the decision makers, and its focus on optimizing the setting of priorities [2, 3].

In response to the widening adoption of MCDA in health care decision making, ISPOR established the MCDA Emerging Good Practices Task Force to provide initial recommendations on how MCDA can best support health care decisions [4, 5]. This Task Force recommends the following steps to combine scientific evidence with stakeholder preferences in any MCDA process: Explicit criteria are selected for alternative courses of action for the health decision problem under consideration. The measured or expected impact of each health care alternative is quantified according to each of the explicitly defined criteria, requiring decision makers to reference, understand, and utilize relevant scientific evidence. To allow meaningful comparisons, the performance metrics are then translated into common-scale scores with uniform increments. For example, quality-adjusted life years (QALYs) and mortality rates by ethnicity might both be converted to a scale from 0 to 100, with higher scores indicating that the health care alternative has a higher impact for that specific criterion. Then, the multiple criteria are weighted based on stakeholder preferences and summed, allowing for a mechanism in which objective health data are melded with subjective values to generate aggregate scores for each health care alternative. Taking explicit account of any uncertainty/limitations in the design and application of the MCDA process, the aggregate scores are interpreted and used to generate a ranking of health priorities that is intended to inform practical and rational priority setting.

One of the preeminent applications of MCDA in global health is the evidence and value: impact on decision making (EVIDEM) framework, created in 2006 to facilitate deliberative and evidence-based multi-criteria health care decision making at the individual and institutional levels [6]. Implementation of the Framework is intended to incorporate accountability for reasonableness (A4R) principles, which state that priority setting should occur in a context of cooperative deliberation and that rationales involved in decision making should be publicly transparent [7]. The EVIDEM framework is designed to raise awareness of the ethical implications of each step of the MCDA process, ranging from the identification of relevant criteria and corresponding evidence to the selection of stakeholders, elicitation of preference weights, and the interpretation of results [8].

Along similar lines, the U.S. National Academies of Sciences, Engineering, and Medicine recently produced a blueprint, a prototype, and use-case scenarios for multi-criteria decision making through the Strategic Multi-Attribute Ranking Tool for Vaccines (SMART Vaccines), to aid priority setting specific to vaccine development, investment, and policy making [9–11]. With over two dozen criteria that extend beyond economic considerations, SMART Vaccines allows decision makers to explicitly incorporate indicators and considerations pertaining to health equity, national security, vaccine delivery, operational management, and scientific and business advancement into vaccine priority setting [12].

Multi-criteria decision analysis developers and users have argued that MCDA’s potential rests on its ability to evolve as both a rigorous instrument and a versatile process in response to diverse stakeholder needs [13, 14]. However, if MCDA is to gain legitimacy and traction in the global health priority setting community, limitations in the assumptions and processes inherent to the development and application of MCDA models must be explicitly addressed.

For instance, selecting comprehensive criteria requires a fully transparent and documented process with input from key stakeholders, such as decision makers (e.g., ministers of health and finance, insurance companies, etc.) and public health beneficiaries (e.g., patients with specific health conditions or members of the general public) [15]. Concurrently, MCDA criteria are more meaningful if the selected criteria do not overlap in their scope and definition. A majority of MCDA demonstrations to date have been criticized for using linear, first-order weighted sums of multiple criteria to generate the final output ranking scores. This process relies on an often-unsupported assumption that the underlying criteria do not overlap and that they are orthogonal and preferentially independent [16, 17]. Even if MCDA models are designed with strictly non-redundant criteria, a simultaneous limitation of such an approach is that it might not extend beyond purely academic/mathematical rationale to take account of more practical and ethical considerations. For example, while MCDA developers and users often attempt to be as comprehensive as possible, they might consider limiting the criteria to an appropriate number based on the availability of impact data and the feasibility of obtaining complete and meaningful stakeholder preferences [17, 18].

Though there is no dominant method for eliciting individual or collective stakeholder preferences, MCDA models and processes are more likely to be incorporated in priority setting activities if they have been developed by researchers and decision makers in close partnership [19, 20]. Similarly, MCDA will be more acceptable if perspectives from the general public are elicited in a representative and meaningful manner [21, 22]. Preference elicitation surveys must be carefully constructed to minimize the cognitive burden on the respondent, while still presenting meaningful questions that are consistent with the full range of criteria and alternatives in a given MCDA framework [23, 24]. To enhance the legitimacy and fairness of these value-driven aspects of MCDA, diverse stakeholder participation will ideally extend beyond a solitary opportunity for input to ongoing “evidence-informed deliberative processes” that facilitate iterative discourse and greater stakeholder understanding throughout every phase of MCDA development and application [25, 26].

Multi-criteria decision analysis can impart greater structure and transparency to priority setting, but effectively leveraging its strengths largely depends on the context in which it is implemented. Developing countries that tend to have implicit and ad hoc priority setting processes are faced with practical barriers such as the dearth of extensive, meaningful data to measure the performance of each alternative according to each criterion [27, 28]. Notwithstanding such limitations, recent evidence suggests that MCDA can provide a structured, objective, and value-based framework in low- and middle-income countries, especially in combination with other approaches such as health technology assessment [15, 29]. The World Health Organization (WHO) recently demonstrated an MCDA application of the WHO-CHOICE methodology, in which key stakeholders from around the world prioritized an extensive list of interventions for the prevention and control of non-communicable diseases, using criteria of cost-effectiveness, feasibility, and equity, as well as health system considerations [30].

How MCDA outputs are interpreted for policy decisions also remains an open question. By definition, MCDA frameworks employ multiple criteria, often with differing units that do not lend themselves to obvious, comparable value improvement thresholds (similar to incremental cost-effectiveness ratios in cost-effectiveness analyses) to account for the opportunity costs in funding decisions [31]. To this end, it has been proposed that existing dollar estimates of willingness to pay for QALYs might be used to create comparable cutoffs for multi-criteria value measures in resource allocation decisions [32]. It has also been suggested that policy entrepreneurs or institutions might be leveraged to outline and oversee the specific goals, designs, rules, ethics, and processes that govern MCDA applications in health [33].

In summary, priority setting in global health typically requires tradeoffs among a variety of clinical, economic, ethical, political, scientific, and social factors that vary across relevant stakeholders. While there remain ethical, conceptual, and empirical challenges to MCDA’s widespread implementation, MCDA has the potential to explicitly identify and account for each of these competing factors in a comprehensive, systematic, and value-driven manner [34].

We would like to thank the editors and managers of the journal *Cost Effectiveness and Resource Allocation* for hosting this *Priority Setting in Global Health* special issue. We would also like to thank the symposium participants for their insightful contributions and commentaries. We would especially like to thank the referees for their detailed review of all the manuscripts. In addition, we are grateful to Mark O’Friel, the Brinson Foundation, and the Payne Family Foundation for their generous financial support for the publication of this special issue. Finally, we would like to thank Mireille Goetghebeur, Guru Madhavan, and Praveen Thokala for their helpful comments on this Introduction.
